# Editorial: Head & Neck Cancer and Esophageal Cancer: From Biosignatures to Therapeutics

**DOI:** 10.3389/fonc.2021.666103

**Published:** 2021-03-19

**Authors:** Victor C. Kok, Cheng-Chia Yu, Jorge A.R. Salvador

**Affiliations:** ^1^ Division of Medical Oncology, Kuang Tien General Hospital Cancer Center, Taichung, Taiwan; ^2^ Department of Bioinformatics and Medical Engineering, Asia University Taiwan, Taichung, Taiwan; ^3^ Institute of Oral Sciences, Chung Shan Medical University, Taichung, Taiwan; ^4^ School of Dentistry, Chung Shan Medical University, Taichung, Taiwan; ^5^ Faculdade de Farmácia da Universidade de Coimbra, Pólo das Ciências da Saúde—Azinhaga de Santa Comba, Coimbra, Portugal

**Keywords:** head and neck squamous cancer cells, biosignatures, cancer biomarker detection, therapeutic developments, immune evasion

Head and neck cancers (excluding thyroid cancer) account for 4.9% of total cancers diagnosed worldwide (1). Optimizing better selection of different therapeutic strategies in head and neck cancer suffers from multiple unmet medical needs because of the critical anatomical site from which cancer arises, chemo- and radioresistance, lacking actionable driver mutations while showing both innate and acquired resistance to immune checkpoint blockade (Kok). Multifaceted cancer management cannot be successful without incorporating measurable and quantifiable biological biomarkers, such as specific enzyme concentration, imaging characteristics, gene phenotype, and metabolomic expression profiles. These biomarkers serve as indicators for cancer risk estimation, prognostication, and treatment monitoring in HNSCC and should be continually investigated.

P16 (INK4A) is an established prognostic biomarker in oropharyngeal cancer, and testing for it has been mandatory in this subgroup of cancer patients (2). Nevertheless, emerging evidence has also supported that P16 has similar prognostication in non-oropharyngeal cancer such as hypopharyngeal, laryngeal, and oral squamous cell carcinoma (OSCC) (3, 4). Over the past few years, rapid advances in genome analysis techniques have opened new possibilities for studying transcriptomes. In 2015, The Cancer Genome Atlas (TCGA) network demonstrated the somatic genome alterations from 279 HNSCC, revealing the genome alterations in P16-positive or smoking-related P16-negative HNSCC, as well as the therapeutic candidate alterations (5). Moreover, increasing evidence has suggested the significant role of aberrantly expressed microRNAs or long non-coding RNAs in head and neck cancer. Furthermore, molecular characterization of HNSCCs will better understand the carcinogenesis and cancer progression process and therapeutic implications. In this Research Topic, we have published studies providing new insights into genetic alterations or novel biological mechanisms underlying the carcinogenesis of head and neck cancer, molecular biomarkers, genomic (e.g., mRNA and long non-coding RNA), or metabolomic or expression profiles for head and neck cancer prognosis, emerging, candidate or novel biomarkers *in silico* or *in vivo* research, biomarkers indicative of resistance to chemotherapeutic or radiation therapy, prognostic or predictive outcome biomarkers, imaging biomarkers of hypoxia or stemness in head and neck cancer using positron emission tomography-computed tomography as an emerging imaging biomarker of occult neck lymph node metastasis in early-stage node-negative tongue cancer.

Unbalanced N^6^-methyladenosine regulation, which affects RNA transcriptional modification, can be carcinogenic, which a paper in our Research Topic has investigated and confirmed its involvement in HNSCC (Zhou et al.). BRAF and its complex component, BCL11A, are known to be altered either as mutation, amplification, or deep deletion in approximately 5% of HNSCC (cBioPortal). Zhou et al. discovered that high levels of BCL11A found in laryngeal SCC tissues, as demonstrated in immunohistochemical staining, were associated with advanced lymph node metastasis and poor prognosis and positively correlated with MDM2 expression. Ambele et al. reported their discovery of a case of oral SCC presenting with high confidence BRAF:p.G469A:c.1406G>C somatic mutation. To investigate the homeobox A cluster (HOXA) gene family as a prognostic factor in laryngeal SCC, Li et al. found that HOXA2 and HOXA4 were downregulated whereas HOXA7 and HOXA9–13 were upregulated in laryngeal SCC. A study from central Taiwan investigated the association between the genotypes, the allelic frequency of long pentraxin 3 (PTX3, also known as TGF14) single-nucleotide variations, and the risk of oral cavity cancer in men (Yeh et al.). In the study, the combined effect of cigarette smoking and PTX3 polymorphism considerably increased the odds of late-stage oral cancer and lymph node metastases (Yeh et al.). Matrix metalloproteinases (MMPs) are implicated in carcinogenesis and cancer progression. It is interesting to note that the transcriptional expression of MMP25 is associated with earlier stage HNSCC, as demonstrated by an *in silico* study using The Cancer Genome Atlas dataset (Liang et al.). The authors have shown that the increased MMP25 expression correlates with increased immune infiltration levels in HNSCC, which significantly activated CD4+ memory T cells (Liang et al.). Resorting to metabolic reprogramming is a cancer hallmark of HNSCC, while increased glycolytic flux is a feature of cancer cells (the Warburg effect). Taking advantage of targeting the glycolysis in cancer cells, scientists have a strong rationale for pursuing glycolytic inhibitors in treating HNSCC. Hexokinase 2 (HK2) is the first enzyme of glycolysis. Li et al. discovered in their study that HNSCC is a glycolytic tumor, and HK2 inhibition does trigger a metabolic shift away from aerobic glycolysis toward mitochondrial metabolism. SMAD4 (also known as Deleted in Pancreatic Cancer-4, DPC4) is a tumor suppressor gene that mediates the TGF-β signaling. Low SMAD4 expression is associated with an approximately 5-fold increase of relapse during follow-up in a cohort of 122 HNSCC patients after adjusting for age, gender, and clinical stage (Lin et al.). Lin et al. leveraging next-generation sequencing analysis based on multiplex-PCR demonstrate that missense SMAD4 mutations could be a potential prognostic biomarker in patients with HNSCC.

Regarding radiological biomarkers development, our Research Topic has two relevant papers investigating the role of positron emission tomography-computerized tomography (PET-CT) scanning in the management of cT1-2N0M0 tongue cancer. In a prospectively enrolled cohort of patients, Zhao et al. found that PET-CT is useful in predicting occult lymph node metastasis; typically, when the SUV max is >9.0, it is significantly associated with worse locoregional control in this subgroup of cT1-2N0 tongue cancer patients. In their retrospective study, Zhu et al. suggested that neck dissection may be avoided when the PET-CT scan reveals no neck lymph node involvement.

We have two bioinformatics research papers using *in silico* methodology to deduce significant gene signatures in HNSCC. Li et al. utilized Weighted Gene Co-expression Network Analysis (WGCNA), differential gene expression analysis, and protein-protein interaction network construction to deduce ten hub genes related to survival from HNSCC. Yang and colleagues leveraged bioinformatics algorithms to establish a long non-coding RNA (lncRNA) signature associated with the prognosis of patients with HNSCC. The investigators ultimately demonstrated an eight lncRNA signature that may be useful in predicting the prognosis; furthermore, they showed that patients with high signature scores might have an abnormal immune function (Yang et al.).

Therapeutic development for HNSCC still requires more research effort to test for novel agents. We have five original articles on trying five chemicals or combinations in controlling HNSCCs. Five independent studies report pre-clinical evaluation of the anti-HNSCC activity of the following agents, hydroxygenkwanin, arsenic trioxide combined with cisplatin, pinostilbene hydrate, psorachromene, and taiwanin E.

Previous studies had revealed that hydroxygenkwanin (HGK), a flavonoid extracted from *Daphne genkwa* Sieb. et Zucc. exhibits anti-cancer effect. Huang and colleagues demonstrated that HGK might be an effective natural product for oral cancer therapy that inhibited cell growth dose-dependently in SAS and OCEM1 cells. They further showed the HGK induced the cell cycle arrest by flow cytometry and inhibited colony formation ability and cell movement. HGK induced intrinsic cell apoptosis pathway and caused cell cycle arrest through p21 activation (Huang et al.).

Hu and colleagues analyzed the inhibitory tumorigenicity of co-treatment with arsenic trioxide and cisplatin on head and neck cancer-initiating cells (HN-CICs) enriched from HNSCC cells. They observed that this drug combination strategy successfully synergized the cell death on HN-CICs with a Combination Index (CI) <1 by Chou-Talalay’s analysis *in vitro* (Hu et al.). In addition, this therapeutic regimen also showed both preventive and therapeutic effects by *in vivo* xenograft assays.

Pinostilbene hydrate (PSH) significantly decreases the activity and expression of MMP-2 and markedly inhibits the abilities of cancer cell migration and invasion (Tseng et al.). Moreover, combined treatment of PSH with ERK1/2 inhibitor (U0126) caused significant elevation of the activity and the expression of MMP-2. Besides, PSH upregulated claudin-1 and E-cadherin expression levels while downregulating vimentin and N-cadherin on two nasopharyngeal carcinoma cell lines (Tseng et al.).

Psorachromene is an isoflavone component isolated from the fruit kernels of *P. corylifolia* L. with anti-inflammatory effects and no previous studies on its anti-cancer activity. Wang and colleagues studied the antitumor effects of psorachromene using cells and animal models of OSCC. Their results revealed that psorachromene significantly inhibited cell proliferation, migration, and invasiveness and increased chemotherapeutic agents’ toxic effects against OSCC cells (Wang et al.).

Taiwanin E is a bioactive compound extracted from *Taiwania cryptomerioides* Hayata. Wang and colleagues studied the anti-cancer effect of taiwanin E against arecoline and 4-nitroquinoline-1-oxide-induced OSCC and elucidated the underlying intricacies. The results showed that taiwanin E significantly attenuated oral cancer cells’ cell viability without significant cytotoxic effects for normal oral cells (N28) (Wang et al.).

In conclusions, we are humble to prepare this editorial after we have formally published the last paper included in this Research Topic because the road in search of the diagnostic, prognostic, and predictive biosignatures for specific settings in HNSCC is still a long way to go and therapeutic development to conquer this debilitating cancer is still flourishing. Nevertheless, colleagues worldwide have strong determinations to do good research on HNSCCs. Through the bit by bit of medical breakthrough, we will someday achieve great success in managing HNSCCs.

## Author Contributions

All authors contributed significantly as guest associate editor and provided feedback on the Editorial. All authors agree on the final manuscript and further publication. All authors contributed to the article and approved the submitted version.

## Conflict of Interest

The authors declare that the research was conducted in the absence of any commercial or financial relationships that could be construed as a potential conflict of interest.
